# Obituary

**Published:** 1869-09-15

**Authors:** 


### OBITUARY.

Resolutions of respect upon the death of Dr. F. O. Earle:

Whereas, The Chicago Medical Society has been deprived of one of its members by the death of Dr. F. O. Earle.

*Resolved*, That we lament his loss. Although he had been with us but a brief time, we had learned to respect him for his gentlemanly deportment and professional ability. Although quite young in the profession of his choice, we cannot but feel that the close application and devotion to the labor of the profession during his stay among us had much to do in determining his early death, which seemed too soon. The profession has lost an earnest worker. To his family and relatives we may be permitted to express our sincere ympathy in their bereavment.

*Resolved*, That a copy of these resolutions be sent to the family, and that they be published in the Medical Journals of this city and in the Medical Record, New York.

R. G. BOGUE, M. D.,

F. D. FITCH, M. D.,

A. H. FULLER, M. D.

Dr. Daniel D. Wait.

Wherras, This Society, with profound regret, has learned of the death of Dr. Wait, one of its oldest and most esteemed members; and

Whereas, It is just and proper for us, who honor him best, to record his name in our archives as one highly respected, not only in eonsequence of his past devotion to the interests of the Society, but as a physician of much intellectual capacity, and one whose character for uprightness and integrity in all its bearings, had but few equals; therefore,

*Resolved*, That by the death of Dr. Wait, this Society has lost one of its oldest and most esteemed members and Chicago a valuable citizen.

*Resolved*, That we evtend our sympathies to the family of the deceased in their heartfelt sorrow during moments of affliction.

*Resolved*, That these resolutions be printed and a copy be sent to his family.

N. S. DAVIS,

G. C. PAOLI,

N. LOVERIN,

*Committee.*

Hiram Wanzer, *Secretary.*

